# Feasibility study of a candidate reference material for ions in PM_2.5_: does commutability matter also for inorganic matrices?

**DOI:** 10.1007/s00216-018-1220-6

**Published:** 2018-07-04

**Authors:** G. Emma, J. Snell, J. Charoud-Got, A. Held, H. Emons

**Affiliations:** European Commission Joint Research Centre, Retieseweg 111, 2440 Geel, Belgium

**Keywords:** Commutability, Aerosols; particulates, Quality assurance; control, Reference materials, Inorganic compounds; trace inorganic compounds

## Abstract

The existing Air Quality Directive 2008/50/EC establishes within the European Union (EU) member states limit values for fine air particulate matter (PM_2.5_) including the possibility to discount natural sources of pollution when assessing compliance with the legislation. In proving this, EU member states shall determine, amongst others, the rural background concentration of some anions (Cl^−^, NO_3_^−^ and SO_4_^2−^) and cations (Na^+^, NH_4_^+^, K^+^, Ca^2+^ and Mg^2+^). To deliver reliable data and to comply with the data quality objectives of the legislation, environmental control laboratories should use certified reference materials (CRMs) to validate or verify the performance of their analytical methods. Since no CRMs for anions and cations in PM_2.5_ are presently available, we present the commutability issues encountered during the first attempt to develop such a material. We demonstrate that a dust, collected in a road tunnel and previously used for the production of two CRMs of a PM_10_-like material, does not behave as an authentic fine particulate matter collected according to EN12341:2014 when measured by an established method proposed by the European Committee for Standardization (CEN/TR 16269:2011). The water-soluble fractions of SO_4_^2−^, NH_4_^+^, K^+^, Ca^2+^ and Mg^2+^ in a PM_2.5_-like candidate CRM produced from that road tunnel dust are only fully extracted after 3 h of sonication and not after 30 min, as stated in the method. Moreover, we found that the particle size of the test material influenced the extraction yield of K^+^, Ca^2+^ and Mg^2+^, suggesting that these ionic species were incorporated in the core of the particles and inaccessible to the extraction procedure. These particular features make the material unsuitable for the measurements of ions with the CEN method. The difference in the extraction time can be seen as a commutability issue and the candidate CRM should be considered as not commutable with routine samples. This demonstrates that commutability studies should not only be considered for clinical CRMs, but also for inorganic CRMs when they are intended to be used to quantify operationally defined analytes.

## Introduction

Airborne particulate matter (PM) cannot be considered as a single pollutant, but rather as a mixture of particles originating from different sources and containing many different chemical species. Particles are emitted into the atmosphere by anthropogenic activities or come from natural events such as volcanic eruption, seismic and geothermal activities, wildfires, sea spray or strong winds that transport particles from dry climate regions. The EU Air Quality Directive 2008/50/EC [1] not only sets limit values for PM, but also gives the possibility for EU member states to provide evidence that the “exceedances” to these limits may be attributable to natural sources. To do this, the Directive requires monitoring of, amongst others, Cl^−^, NO_3_^−^, SO_4_^2−^, Na^+^, NH_4_^+^, K^+^, Ca^2+^ and Mg^2+^ in PM_2.5_ (fraction of PM which passes through a size-selective inlet with a 50% cut-off efficiency at 2.5-μm aerodynamic diameter [2]), collected at rural background locations. Hence, in 2011, the European Committee for Standardization (CEN) released a procedure for the measurements of inorganic ions in PM_2.5_ (CEN/TR 16269:2011). Although this is not a standard method and there is no legal obligation to use it, European environmental control laboratories widely employ this procedure since it is based on well-established measurements from the Cooperative Programme for Monitoring and Evaluation of the Long-range Transmission of Air Pollutants in Europe (EMEP) organised under the auspices of the United Nations Economic Commission for Europe. To guarantee the accuracy of these measurements and compliance with the data quality objectives laid down in Directive 2008/50/EC, environmental control laboratories have to use adequate quality assurance and quality control tools and should ensure that all measurement results are traceable in accordance with ISO/IEC 17025. This can be achieved by using certified reference materials (CRMs) when validating or assessing performance of the analytical methods employed [3]. Unfortunately, neither of the two existing CRMs of PM_2.5_ commercially available (NIST SRM 2783 and NIST SRM 2786) are certified for the ions listed in the Air Quality Directive [4, 5]. Therefore, a new CRM is currently under development at the European Commission Joint Research Centre (JRC) [6]. In contrast to other PM materials loaded on filters such as NIST SRM 2783, NIST RM 8785 and CRM SL-MR-2-PSF-01 [7–9], the new candidate CRM will be produced as a fine powder. In such a physical form, materials are usually simpler to homogenise, more stable and easier to store and transport [4, 10]. In addition, the uncertainties of the certified values are expected to be lower than those for filter-based CRMs because the variability of the deposition process is absent. An alternative production procedure was developed and is described elsewhere [6]. The material was found to be homogenous and stable, and is currently undergoing characterisation.

When developing a CRM, it is important to ensure stability and homogeneity of the certified parameters in the material, as well as to guarantee that the material behaves like a real sample when using established analytical methods, in other words, that it is commutable [4]. Commutability is a property of reference materials (RMs) that define the equivalence of the mathematical relationships amongst the results of different measurement procedures for an RM and for representative samples of the type intended to be measured [11]. This concept was first used in the early 1970s to describe the comparability of enzyme activity measurements between reference and control materials to authentic clinical samples [12, 13]. Following this, many articles published in clinical journals have emphasised the importance of validating the commutability of RMs, especially if used as calibrators (for examples, see references [14–18]). CRMs for inorganic analysis are normally not considered to be affected by this problem as methods are typically optimised to fully extract the analytes of interest. However, in some occasions, laboratories need to follow established procedures for method-specific (also called operationally defined) determinations, such as leaching, and commutability of the CRMs can, therefore, become an issue. For instance, the procedure described in CEN/TR 16269:2011 foresees a 30-min water extraction in an ultrasonic bath to fully extract the inorganic ions from PM_2.5_. In this case, the analyte is only the water-soluble fraction of these ions under the prescribed experimental conditions and commutability should be checked carefully.

In this paper, we report the results of a feasibility study of the candidate CRM of PM_2.5_ currently under development which is intended to be certified for all the ions listed in the European Air Quality Directive. Particular attention will be given to the commutability with a view to transfer this concept also out of the clinical environment.

## Materials and methods

The starting material for the PM_2.5_ candidate CRM production was the same as that used to produce ERM-CZ100 and ERM-CZ120, two CRMs of a PM_10_-like matrix (fraction of suspended PM which passes through a size-elective inlet with a 50% cut-off efficiency at 10-μm aerodynamic diameter [2]) released in 2010 by the JRC. These materials, certified for the content of PAHs and heavy metals, respectively, were produced by jet milling a sieved road dust, collected from the walls and sidewalks of the road tunnel Wisłostrada in Warsaw, Poland [19].

The PM_10_-like material, which was mixed and stored at − 20 °C, was further jet-milled using a Hosokawa Alpine Picoline jet mill, operated with a classifier wheel revolving at 30,000 rpm in order to obtain a finer powder. The particle size distribution of the PM_2.5_-like candidate CRM obtained after the milling process was measured by a low-angle laser light diffraction technique via suspending the particles in 2-propanol under constant stirring in a 50-mL cuvette and using a Sympatec Helos device.

Pall Tissuquartz QUAT-UP 47-mm filters were used to prepare simulated PM_2.5_ quartz filter samples, applying a similar technique to that recently proposed in the literature for the development of the CRM LNE SL-MR-2-PSF-01 [9]. Briefly, 25 mg of dust was pressed on the filters until complete penetration into the fibres by using an agate pestle.

Authentic air-sampled PM_2.5_ samples on Teflon filters were collected by the Flemish Institute for Technological Research (VITO) during a campaign that took place between December 2011 and January 2012 near the R1 Antwerp ring road. Samplers were placed at a distance of less than 100 m from the road. Therefore, the dust collected represents an urban background with a significant contribution of traffic emissions.

Water-extractable ions were measured by ion chromatography (IC) using a Metrohm 850 Professional IC in the PM_10_-like starting material, the PM_2.5_-like candidate CRM, the simulated PM_2.5_ quartz filter samples and the authentic PM_2.5_ filter samples. For the two powder materials, about 25 mg was extracted in 25 mL of water in an ultrasonic bath for 30 min, according to CEN/TR 16269:2011, and for different extraction times. Solutions were then passed through 0.45-μm PTFE filters and analysed directly by IC. For the simulated PM_2.5_ filter samples, each filter was immersed in 25 mL of water in a sample tube and exposed in an ultrasonic bath for 30 min as well as for different periods of times. Once again, the extracts were analysed by IC after filtering them on 0.45-μm PTFE filters. Blank filters were also analysed by the same procedure. For the authentic PM_2.5_ filter samples, each 47-mm diameter Teflon filter was cut into two or four similar pieces using ceramic scissors and then extracted in 10 mL of water for 30 min, as described in the CEN method, and by applying different extraction times. As before, the solutions were passed through 0.45-μm PTFE filters and analysed by IC. The conditions of the ion chromatography are summarised in Table 1. Standard solutions for calibration curves and quality control samples were prepared from Merck (anions) and Fluka (cations) CRMs. IC eluents were prepared from analytical grade solution purchased from Sigma-Aldrich. MilliQ ultrapure water was used to prepare all the solutions and to extract ions from the PM materials.

**Table 1:** IC conditions for all the experiments performed during this study

Parameter	Value
Eluent	3.2 mM Na_2_CO_3_/1.0 mM NaHCO_3_ (anions) 1.7 mM PDA/1.7 mM HNO_3_ (cations)
Flow	0.7 mL/min (anions) 0.9 mL/min (cations)
Columns	Metrosep A Supp 5 - 250/4.0 + Metrosep A Supp 4/5 Guard/4.0 Metrosep C6 - 150/4.0 + Metrosep A Supp 4/5 Guard/4.0
Temperature	30 °C
Sample loop	250 μL
Injection volume	20 μL (anions) 200 μL (cations)
Suppressor	0.1 M H_2_SO_4_ (for anions only)
Detector	Conductivity detector

## Results and discussions

**Fig. 1:**
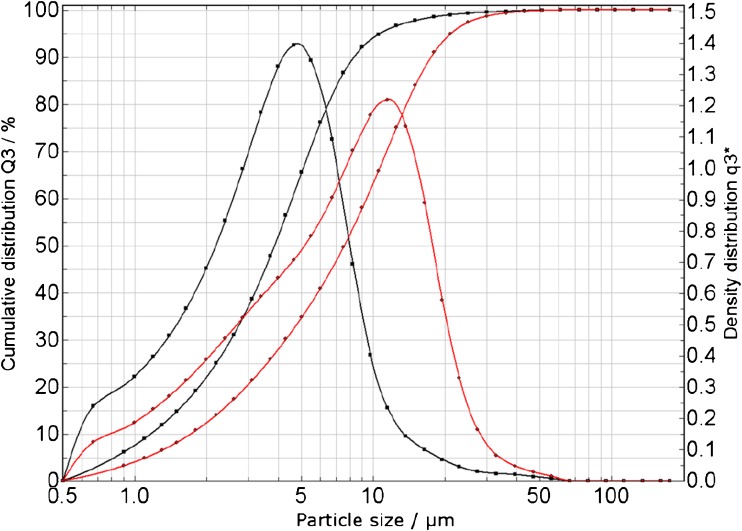
Particle size distribution obtained for the PM_10_-like starting material (red lines) and the PM_2.5_ candidate CRM (black lines)

Applying the procedure described in the section above, the PM_10_-like starting material was successfully milled into a finer powder. In Fig. 1, the cumulative distribution of the samples’ particle size of both materials (PM_10_-like and PM_2.5_-like candidate CRM) is presented. As can be seen, 90% of the PM_2.5_-equivalent sphere particle diameter is below 8.5 μm and that 50% is below 3.5 μm. Bearing in mind that airborne particles have different irregular shapes and cannot be easily described by spheres, we consider that the particle size distribution could reflect the particle sizes typical for authentic PM_2.5_.

**Fig. 2:**
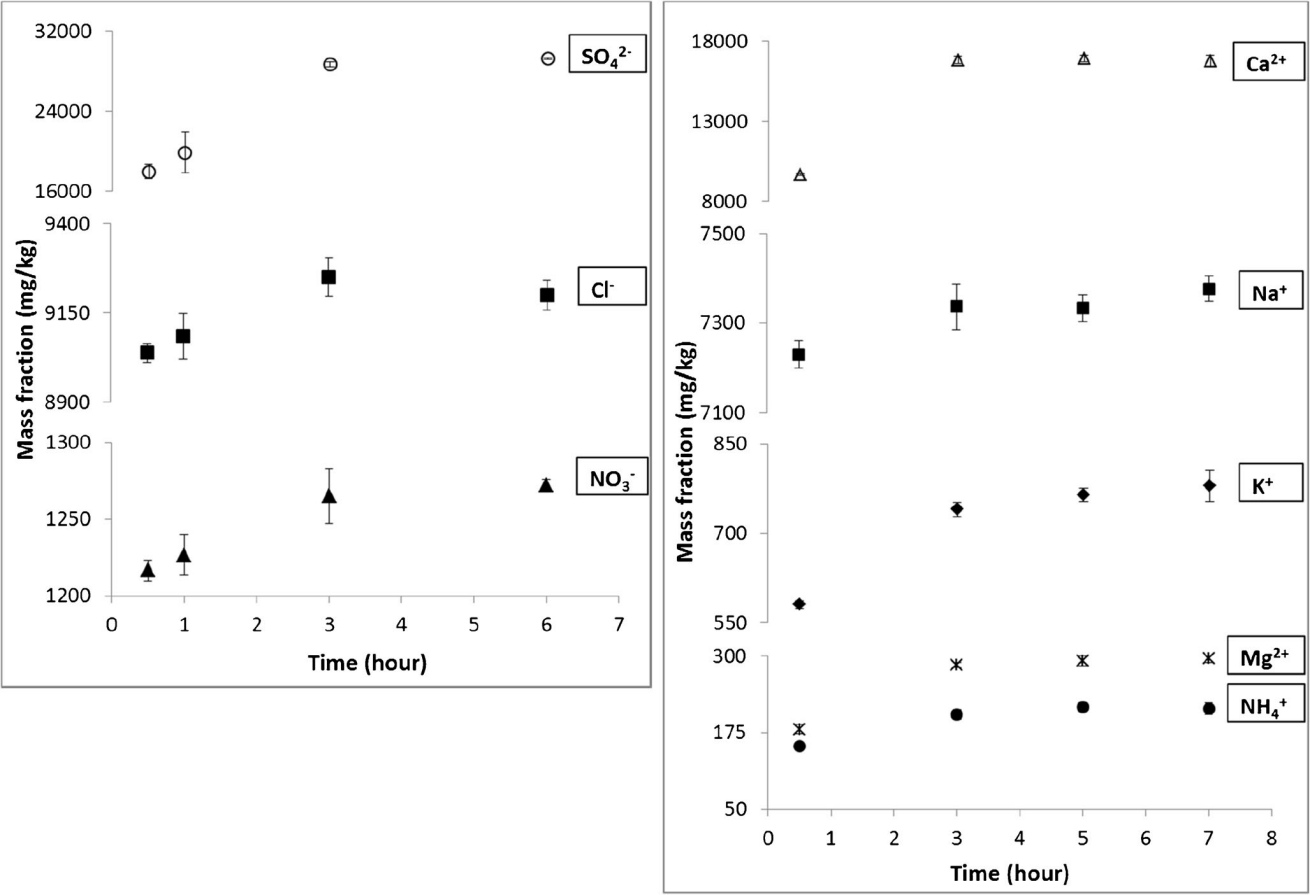
Mass fraction of water-extractable anions (left) and cations (right) of different extraction times with the PM_10_-like starting material

Once processed, the candidate CRM must undergo a series of studies in order to assess homogeneity and stability and to certify the mass fraction of, in this particular case, inorganic ions. Unfortunately, feasibility studies performed by applying the CEN reference method (CEN/TR 16269:2011) showed that the sample extraction procedure does not work as described in the method. For both the PM_10_-like starting material and PM_2.5_-like candidate CRM, 30 min of sonication is not sufficient to completely extract the water-soluble fraction of all the ions. In fact, as can be seen in Fig. 2, full extraction of SO_4_^2−^, NH_4_^+^, K^+^, Ca^2+^ and Mg^2+^ from the PM_10_-like material is achieved only after 3 h. Similar results were also obtained for the PM_2.5_-like candidate CRM. Moreover, it was observed an increment in the extraction yield for K^+^, Ca^2+^ and Mg^2+^ proportional to the reduction in the particle size: after 30-min sonication, the extraction efficiency of these elements in the PM_2.5_ candidate CRM increased to 8.6, 11.2 and 33.1% for K^+^, Ca^2+^ and Mg^2+^ respectively.

**Fig. 3:**
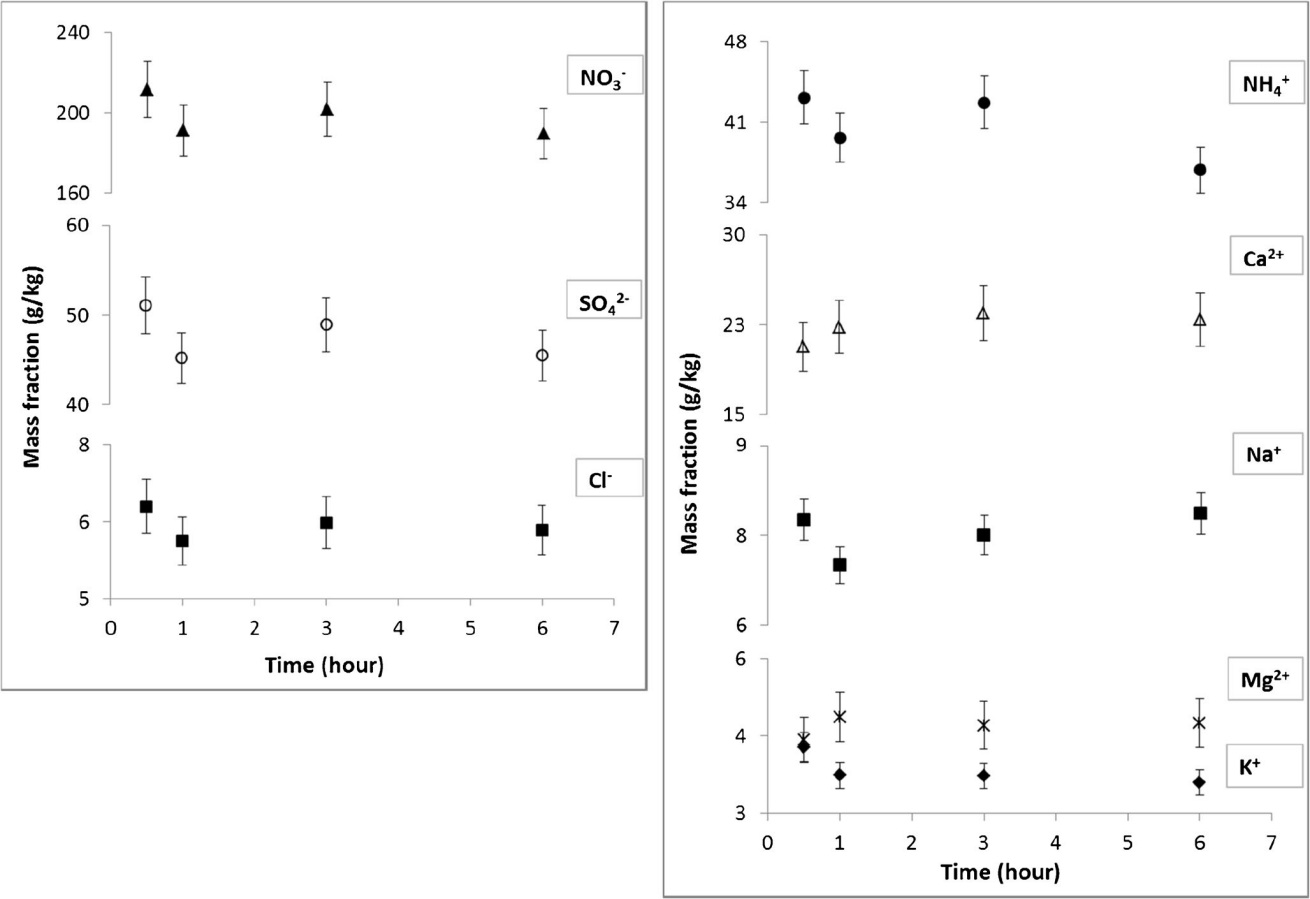
Water-extractable ion mass fraction from authentic PM_2.5_ Teflon filter samples extracted at different extraction times. The error bars refer exclusively to the within filter heterogeneity contribution to the total uncertainty established in separate experiments under repeatability conditions

To verify whether the extraction time is an issue also for authentic samples, atmospheric PM_2.5_ filter samples were analysed. As described in the experimental part, a round Teflon filter was cut into four pieces. The extraction was performed on single quarters for 30 min, 1 h, 3 h and 6 h to check the influence of the extraction time. It was necessary to use a single filter for the comparison, as filter samples were collected at different times and locations, and the amounts of ions varied widely between samples. The results for each extraction time can be compared in Fig. 3. The experiment was repeated with two additional filters to calculate a mean deviation between the time points. These mean deviations are listed in the second column of Table 2. To be sure that any difference between time points was not simply due to inhomogeneity between the quarters of each filter, two other filters were quartered and extracted with the same extraction time (3 h). The mean deviations observed within each filter are listed in the first column of Table 2 and graphically represented as the vertical bars on each time point in Fig. 3. Finally, another set of filters was cut into two, and each half was extracted for either 30 min or 3 h. This confirmed that there was no significant difference between the level of deviation observed with different extraction times (data not shown).

**Table 2:** Comparison of the average of the RSDs from replicate measurements on authentic PM_2.5_ filter samples extracted applying the same extraction time (within filter homogeneity) and different extraction times, respectively. *F* is the results of the statistical test where the variance of the measurements (*s*^*2*^*)* is compared. *F*_tab_ is the *F* critical value at 95% confidence level (CL)

Ion	Same extraction timeAverage RSD (%)	Different extraction times Average RSD (%)	$F=\frac{s_1^2}{s_2^2}$	*F*_tab_ (95% CL)
Cl^−^	9.5	6.2	3.45	3.60
NO_3_^−^	6.6	7.9	1.34	3.01
SO_4_^2−^	6.2	6.0	1.03	3.60
Na^+^	4.4	4.6	1.03	3.01
NH_4_^+^	5.4	7.0	1.71	3.01
K^+^	5.8	6.1	1.10	3.01
Ca^2+^	9.9	10.9	1.01	3.14
Mg^2+^	8.8	3.9	7.37	4.15

The inhomogeneity of ion distribution of filter samples is evident when comparing the error bars for each data point between Figs. 2 and 3. To compare the variance between measurements with the same extraction time and with different extraction times, an *F* test was performed on data normalised to the average concentrations of ions in each sample. At the 95% confidence level, the variance between the two sets of observations was not different with the exception of the data for Mg^2+^, which showed lower variance in measurements at different extraction times. This shows that there was no significant trend attributable to the extraction time and 30 min was enough to obtain complete dissolution of all water-soluble ions in authentic PM_2.5_ filter samples. Therefore, when applied in our laboratory, the CEN method was clearly fit for purpose for real atmospheric PM_2.5_ filter samples.

The variation observed for the results of all the ions is in line with that expected for different measurements on different portions of the same filter, when considering the field sampling uncertainty, estimated at about 5% (variation in air sampler flow rates and sample volume, the deposit density on the filter and possible sample loss and contamination in addition to instrumental variance [20]). In contrast, different replicates of the PM_2.5_-like candidate CRM extracted for the same time (of 3 h), and IC standards all measured under repeatability conditions on different days, showed significantly lower RSDs, below 2 and 1%, respectively. As the lower RSD of the standards demonstrates that the instrumental variance is negligible, this also confirms that the variance of measurement results on authentic atmospheric samples is mainly due to their heterogeneity, while the PM_2.5_-like candidate CRM is clearly more homogenous.

**Fig. 4:**
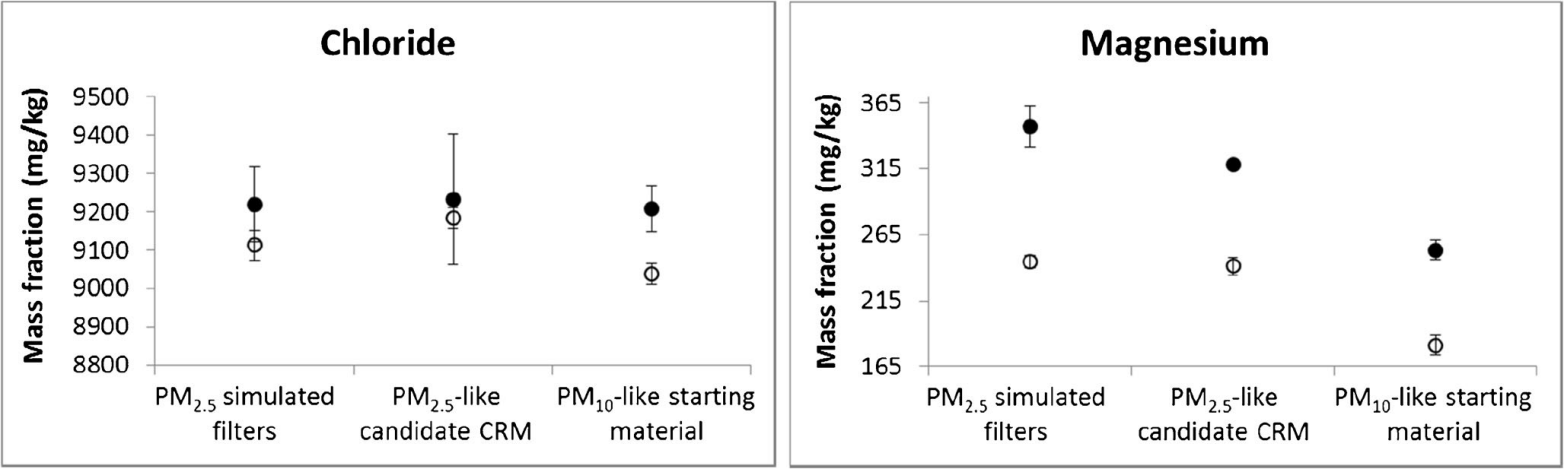
Mass fraction of Cl^−^ and Mg^2+^ in PM_10_-like starting material, PM_2.5_-like candidate CRM and PM_2.5_-simulated filter samples after 30-min (open circles) and 3-h extraction (filled circles) as an example of different behaviours. Error bars are the standard deviation of different measurement replicates

The physical form in which the material is presented can also influence the outcome of the extraction procedure of the CEN method. Therefore, to have the same physical form as real samples, the PM_2.5_-like candidate CRM was loaded onto quartz fibre filters following the procedure recently proposed by the *Laboratoire national de métrologie et d’essais* (LNE) [9] and described in the section above. The two powder materials (PM_10_-like starting material and PM_2.5_-like candidate CRM) together with the simulated PM_2.5_ filter samples were again extracted for 30 min and 3 h and analysed under repeatability conditions. Two examples of the results are shown in Fig. 4. Na^+^ and NO_3_^−^ behave like Cl^−^ for which complete extraction is achieved in 30 min. In contrast, as observed for the PM_10_-like starting material and the PM_2.5_-like candidate CRM, the water-soluble fraction of SO_4_^2−^, NH_4_^+^, K^+^, Ca^2+^ and Mg^2+^ was only fully extracted after 3 h. Therefore, loading the powder on filters did not increase the extraction efficiency, and simulated PM_2.5_ filter samples and the PM_2.5_-like candidate CRM in the physical form of powder are comparable as far as the analytical procedure is concerned.

Hence, the reason behind the difficulty of extraction is not related to the material presentation. One possibility is that the characteristics of the material were causing different extraction behaviours during analysis by the CEN method. As already explained, the material was originally a road tunnel dust and, after the first sieving step, still contained particles up to 180 μm [19]. Since sieves were not able to separate PM_10_ from bigger particles, the PM_10_-like CRM was obtained by using a jet mill machine which enables to both reduce the particle size through high-velocity collisions between particles in a turbulent air flow and to separate them. The same procedure was also adopted here to obtain the PM_2.5_-like candidate CRM. For the purpose of the two previous CRM production (ERM-CZ100 and ERM-CZ120), the use of the jet mill technique was considered fit for purpose since heavy metals and PAHs are determined after complete digestion of the samples or full extraction of the analytes in the appropriate organic solvent. On the contrary, the determination of ions in PM_2.5_ is method-defined and it is consequently possible that only a portion of the total ion content is actually measured. Since, as already reported, PM_2.5_, PM_10_ and coarser particles have a different chemical composition [21], the use of a milling technique to produce a material intended to be certified for ions could pose some problems. In fact, although the different fractions of PM are partially overlapping, it has been demonstrated that mineral dust and sea salt contribute more to PM_10_ or coarser fractions of air particulate, while NH_4_^+^, NO_3_^−^, SO_4_^2−^ and organic and elemental carbon are more present in the PM_2.5_ fraction [22, 23]. It is therefore obvious that the chemical composition of the candidate CRM under investigation cannot be similar to that of the authentic atmospheric PM_2.5_ as particles were created by grinding the coarse dust material. In addition, since the raw material was collected in a city road tunnel, it would not be surprising if it may contain additional organic matter from vehicle exhaust systems or pieces of tyre rubber usually not present in the atmospheric PM_2.5_ fraction. This excess of hydrophobic substances together with the expected presence of a high concentration of mineral dust provides a likely explanation for the difficulties in extracting some ions. Those could either be hidden in the mineral dust or masked by the excess of the organic matter. This would also explain why the extraction efficiency increases for some ions with decreasing particle size: certain ions enclosed in the mineral dust could become accessible for the extraction solvent by breaking the particles, while others simply need more time to dissolve in water. Therefore, the candidate material behaved differently than authentic atmospheric samples because of its different composition and a different distribution of various ions under investigation in the particles. This difference can also be considered as a good example of a commutability issue for the PM_2.5_-like candidate CRM.

While different ways to assess commutability of RMs exist in the clinical chemistry environment [11, 15], they all use descriptive statistics or some form of regression analysis to compare the relationship amongst methods. One of the most applied approaches is to verify that the (C)RM lay within the prediction interval, usually set at 95% of the Deming regression used as a best-fit for a real clinical sample dataset analysed with two different methods (normally, a reference method vs. the method under investigation). It should not be surprising that, for example, two immunoassay methods give different responses when analysing real clinical samples; this is because they are based on reactions with different parts of the protein of interest. In any case, the (C)RM, if commutable, would show the same behaviour as real clinical samples when used as calibrator for the selected analytical methods.

However, in the case of this investigation, a Deming regression has two limitations. Firstly, the concentrations of ions in the PM_2.5_-like candidate CRM and authentic PM_2.5_ filter samples differ, except for Ca^2+^, by orders of magnitude. Hence, measurement results of the PM_2.5_-like candidate CRM are outside the range described with the dataset from real samples. Secondly, the measurement uncertainties are not comparable since, as we have demonstrated, authentic filter samples are affected by higher within-unit (within-filter) inhomogeneity. For the analytical tasks discussed here, the analytes are operationally defined by CEN/TR 16269:2011 which can be seen as a reference method. Using the same method, but changing the extraction time from 30 min to 3 h results, to a certain extent, in a new method. As demonstrated by this study, 3 h was in fact needed to bring the whole water-soluble fraction of several ions from the PM_2.5_-like candidate CRM into solution. As summarised in Table 3, the ion mass fraction in the extract was definitely higher after 3 h: 53% for SO_4_^2−^, 17% for NH_4_^+^ and K^+^, 49% for Ca^2+^ and 32% for Mg^2+^. Hence, the reference method and the new one behaved differently when the PM_2.5_-like candidate CRM was measured. On the other hand, the results of atmospheric PM_2.5_ samples are not affected by the different extraction times. Therefore, it can be concluded that the CEN/TR 16269:2011 method is indeed fit for purpose for measurements of ions in authentic air-sampled PM_2.5_ filter samples, but not for the same measurements in the candidate CRM. Alternatively, it could be stated that, according to the definition of commutability, the representative samples of the type intended to be measured and the candidate CRM behave differently. Under these circumstances, the PM_2.5_-like candidate CRM under investigation cannot be considered commutable with routine samples.

**Table 3:** Ion content extracted after 30 min and 3 h from PM_2.5_-like candidate CRM samples

Ion	30-min extraction (mg/kg)	3-h extraction (mg/kg)	Difference (3 h−30 min)
Cl^−^	9183 ± 26	9233 ± 170	0.5%
NO_3_^−^	1272 ± 8	1304 ± 25	2.6%
SO_4_^2−^	19,026 ± 422	29,049 ± 387	52.7%
Na^+^	7311 ± 22	7394 ± 112	1.1%
NH_4_^+^	147 ± 1	172 ± 4	17.2%
K^+^	631 ± 24	737 ± 7	16.7%
Ca^2+^	10,764 ± 261	16,083 ± 252	49.4%
Mg^2+^	241 ± 7	318 ± 3	32.0%

## Conclusions

It has been confirmed here that for the analysis of authentic PM_2.5_ filter samples, a full extraction of all the ions under investigation is obtained within 30 min and that the procedure described by CEN is fit for purpose for the determination at urban background sites. However, it was found, when investigating whether a jet-milled dust obtained from a road tunnel was a suitable candidate material for a PM_2.5_ CRM, that this material was not commutable to the measurement of authentic PM_2.5_ samples by CEN/TR 16269:2011 due to its different composition. An alternative procedure was therefore developed and it is described elsewhere [6].

However, this paper highlights the importance of assessing the suitability of RMs for method validation, whenever the candidate RM is intended to be used for operationally defined measurands. Therefore, commutability studies, rarely considered for tasks other than clinical measurements, may also be necessary for inorganic analysis and this study clearly demonstrated this need.
